# Association analysis between constructed SNPLDBs and GCA effects of 9 quality-related traits in parents of hybrid rice (*Oryza sativa* L.)

**DOI:** 10.1186/s12864-019-6428-0

**Published:** 2020-01-09

**Authors:** Moaz S. Eltahawy, Nour Ali, Imdad U. Zaid, Dalu Li, Dina Abdulmajid, Lal Bux, Hui Wang, Delin Hong

**Affiliations:** 1 Nanjing Agricultural University, Nanjing, 210095 China; 2 State Key Laboratory of Crop Genetics and Germplasm Enhancement, Nanjing Agricultural University, Nanjing, 210095 China; 3 Agronomy Department, Faculty of Agriculture, Zagazig University, Sharqia, 44519 Egypt; 4 Laboratory of Crop Genetics and Germplasm Enhancement, Field Crops Research Department, Agricultural Faculty, Damascus University, Damascus, Syria; 5 Rice Research and Training Centre, Field Crops Research Institute, Agricultural Research Centre, Kafr El-Sheikh, 33717 Egypt

**Keywords:** Hybrid rice, Combining ability of parents, Single nucleotide polymorphism linkage disequilibrium blocks, Association analysis, Quality traits

## Abstract

**Background:**

The general combining ability (GCA) of parents in hybrid rice affects not only heterotic level of grain yield and other important agronomic traits, but also performance of grain quality traits of F_2_ bulk population which is the commodity consumed by humans. In order to make GCA improvement for quality traits in parents of hybrid rice by molecular marker assisted selection feasible, genome-wide GCA loci for quality traits in parents were detected through association analysis between the effects of GCA and constructed single nucleotide polymorphism linkage disequilibrium blocks (SNPLDBs), by using unhusked rice grains harvested from F_1_ plants of 48 crosses of Indica rice and 78 crosses of Japonica rice. GCA-SNPLDBs association analysis.

**Results:**

Among the 8 CMS and 6 restorer lines of *indica* rice subspecies, CMS lines Zhenpin A, Zhenshan97 A, and 257A, and restorers Kanghui98, Minghui63 and Yanhui559 were recognized as good general combiners based on their GCA effect values for the 9 quality traits (brown rice rate, milled rice rate, head rice rate, percentage of chalky grains, chalky area size, chalkiness degree, gelatinization temperature, gel consistency and amylose content). Among the 13 CMS and 6 restorer lines of *japonica* rice subspecies, CMS 863A, 6427A and Xu 2A, and restorers C418, Ninghui8hao and Yunhui4hao showed elite GCA effect values for the 9 traits. GCA-SNPLDB association analysis revealed 39 significant SNPLDB loci associated with the GCA of the 9 quality-related traits, and the numbers of SNPLDB loci located on chromosome 1, 2, 3, 4, 5, 8, 9, 11 and 12 were 1, 4, 3, 9, 6, 5, 5, 4 and 2, respectively. Number of superior GCA alleles for the 9 traits among the 33 parents ranged from 1 to 26.

**Conclusions:**

Thirty-nine significant SNPLDBs loci were identified associated with the GCA of 9 quality-related traits, and the superior SNPLDB alleles could be used to improve the GCA of parents for the traits in the future by molecular marker assisted selection. The genetic basis of trait GCA in parents is different from that of trait itself.

## Background

Rice (*Oryza sativa* L.) is a crucial staple crop for more than half of the world population. Recently, due to the increase in their living standards, people started to demand high-quality rice, including high eating and cooking quality, with various preferences in different geographic regions. Breeding researcher more centered on enhancing the quality of rice to cope with the demanded quality standards of direct consumers, and the other various commercial uses. Grain quality in rice is determined through many factors, e.g., nutritional value, grain appearance, cooking and eating quality. Among 117 rice-growing countries, hybrid rice breeding technologies have been adopted by 27 countries. Grain quality of hybrid rice has its speciality since the commodity consumed by people is a F_2_ bulk population.

From a commercial perspective, the key to gain high grain quality from hybrid rice depends on the choice of parental material. The prime initiative of rice breeders for developing superior hybrid rice cultivar is to choose suitable mating parents [5]. These parental characteristics are heritable and were able to appear in the F_1_ generation. The combining ability is the basic breeding tool for identification of prospective parents of hybrid cultivars for both yield and quality traits. Generally, combining ability is an estimation and prediction of parental values relayed on their developed offspring performances [34]. Typically, evaluation of inbred parents and crosses for GCA following the traditional plant breeding methods are laborious, tedious and time-consuming [33]. In addition, as the number of parents involved in combining ability manipulation increased, their hybrids affected the feasibility of the experiment [3]. Many studies based on association analysis between combining ability and markers also revealed genomic loci significantly found associated with the combining ability of parental traits [13, 16, 18, 19, 22, 27, 37]. Several SSR marker loci associated with the CA of quality traits have been published. However, these studies were confined to SSR markers. Thus far, no SNP-based analyses were reported to discover SNPLDB locus/loci associated with the GCA of parental quality traits in rice.

In this study, to increase the power of association analysis for discovery of GCA loci of quality-related traits, we suggest a grouping of identified SNPs into haplotype blocks (SNPLDBs). The principle of blocking was determined based totally on tightly linked genetic loci. SNPs are usually located close to each other and trend to move together. In general, genetic loci located more adjutants to others on a chromosome had strong LD compared to those present distantly. The construction of SNPLDBs and treating them as an independent unit (marker), we are minimizing the number of assumptions being tested and thus relaxing the strict criteria for gaining maximum significance of association analysis. Merging SNPs together in a proper way extends the dimension of association analysis. Furthermore, if there are multiple independent SNPs, by considering their joint effect, we will have the power to detect this joint effect on the trait. Recently, the LD blocks-based SNPLDB marker have been proposed for association analysis and showed practical utility value in the experiments of plant breeding [25, 40].

Here, we treated the constructed SNPLDB as a marker and examined in the associations with the values of GCA for 33 parents of hybrid rice for 9 quality-related traits, using the single factor ANOVA method of marker-trait association. The sequence data were obtained by performing genotyping by sequencing of parental genomes, whereas, the GCA effects were estimated by evaluation of developed hybrids.

The objectives of our study were: (1) to evaluate parents of hybrid rice for GCA effect of quality traits; (2) to associate SNPLDB with the parents GCA to determine genome-wide GCA loci and superior SNPLDB alleles related to grain quality traits; (3) to predict combinations that can improve GCA effect values of parents for the quality traits through pyramiding or substituting SNPLDB alleles.

## Results

### Performance of 9 quality traits of F_2_ bulks in two sets of NCII combinations

The mean performances of 9 quality-related traits in 48 hybrids obtained from 8 indica rice CMS lines crossed with 6 indica restorers are presented in Additional file 1: Table S1. Among the 48 Indica developed crosses, the highest brown rice rate (86.3%), gelatinization temperature (6.2ASS) and amylose content (23.9%) in addition to the least chalkiness degree (1.6%) were observed in Zhenshan97A × Kanghui98. The cross between CMS Yuetai A and restorer Yanhui559 recorded the highest milled rice rate (76.2%) and head rice rate (69.5%), while, The least percentage of chalky grains (36.0%), chalky area size (16.7%) and gel consistency (37.5 mm) was detected in 256A × Zhenhui084.

The mean performances of 9 quality-related traits in 78 hybrids obtained from 13 japonica rice CMS crossed with 6 japonica restorers are presented in Additional file 2: Table S2. Among the 78 japonica developed crosses, the mean performance of Wuyujing3A × Ninghui8hao showed the highest brown rice rate (83.8%), milled rice rate (73.7%), head rice rate (67.1%) and gel consistency (62.0 mm) in addition to the least chalkiness degree (1.6%). The cross between CMS 731A and restorer Yanhui R50 recorded the least percentage of chalky grains (30.5%), chalky area size (11.5%) and gelatinization temperature (1.1ASS), whereas, the least amylose content (9.6%) was detected in Liuyan 189A × Yanhui R50.

### Estimations of GCA effects of indica rice CMS and restorer lines

In our study, the effect values of GCA for CMS and restorer lines in indica rice varied significantly for 9 quality-related traits. The 14 parents (8 CMS lines + 6 restorer lines) of indica rice set showed both positive and negative GCA effect values. For example, the GCA of II-32A showed a negative effect for chalky area size, percentage of chalky grains, chalkiness degree and amylose content, but positive effect on head rice rate, milled rice rate, brown rice rate, gel consistency and gelatinization temperature. Among the 8 indica CMS lines, the GCA effects of CMS Zhenpin A showed maximum positive values for all traits (Table 1). Also, the CMS Zhenshan97A was observed to be good general combiner for chalky area size, percentage of chalky grains, chalkiness degree and gel consistency.

**Table 1:** Effect values of GCA of Indica CMS and restorer lines for 9 quality-related traits

CMS lines	BRR (%)	MRR (%)	HRR (%)	PCG (%)	CAS (%)	CD (%)	GT (ASS)	GC (mm)	AC (%)
256A	−2.7d	-2.4d	-2.2e	-2.8e	-2.5e	0.08a	− 0.53 g	−2.5c	− 2.4e
Zhenpin A	2.1a	1.8a	1.7a	2.5a	2.2a	−0.06 h	0.78a	1.9a	2.2a
257A	0.8b	0.7b	0.6bc	1.3bc	1.2b	−0.04f	0.14c	0.6a	1.3b
II-32A	0.5b	0.4b	0.4c	−0.2d	−0.1c	− 0.01d	0.14c	0.4a	−0.1c
Zhenshan 97 A	1.1b	0.9b	0.8b	2.1ab	1.8a	−0.05 g	0.18b	1.0a	1.3b
Yuetai A	−1.2c	−1.0c	−0.9d	−2.9e	−2.6e	0.07b	−0.31e	− 1.1b	− 2.4e
You 1A	−1.3c	− 1.2c	− 1.0d	− 0.8d	− 0.7d	0.03c	−0.42f	− 1.1b	− 0.6d
Zhong 9A	0.8b	0.7b	0.6bc	0.8c	0.7b	−0.03e	0.03d	0.7a	0.8b
Restorers									
Minghui 63	0.5b	1.3a	2.2a	2.2a	2.5a	−0.09f	0.14c	1.4a	2.5a
Zhenhui 084	−1.6c	−1.3b	−0.9b	−0.4bc	− 0.3d	0.01c	− 0.21d	− 1.4b	−0.2d
Yanhui 559	1.5a	1.6a	2.0a	0.5b	0.6c	−0.02d	0.41a	1.6a	0.6c
Huizi 04	−1.2c	−1.7b	−1.9c	−0.7c	− 1.0e	0.04b	−0.31e	− 1.8b	−0.9e
Hui 9368	−1.1c	− 1.8b	−3.4d	− 3.2d	− 3.2f	0.11a	− 0.34f	− 1.7b	− 3.1f
Kanghui98	2.0a	1.9a	2.0a	1.6a	1.5b	−0.05e	0.30b	2.0a	1.1b

The Indica CMS and restorer lines trail by alphabets are significantly different at *P* < 0.01

Among the 6 indica restorer lines, Minghui63 had maximum GCA effect values for head rice rate, chalky area size, percentage of chalky grains, chalkiness degree and amylose content; Kanghui98 showed maximum positive GCA values for milled rice rate, brown rice rate and gel consistency; Yanhui559 showed maximum positive GCA value for gelatinization temperature (Table 1). In terms of elite parental lines, the CMS Zhenpin A, Zhenshan97A and restorer Minghui63, Kanghui98 and Yanhui559 had the most favorable GCA effects for the studied traits.

**Fig. 1:**
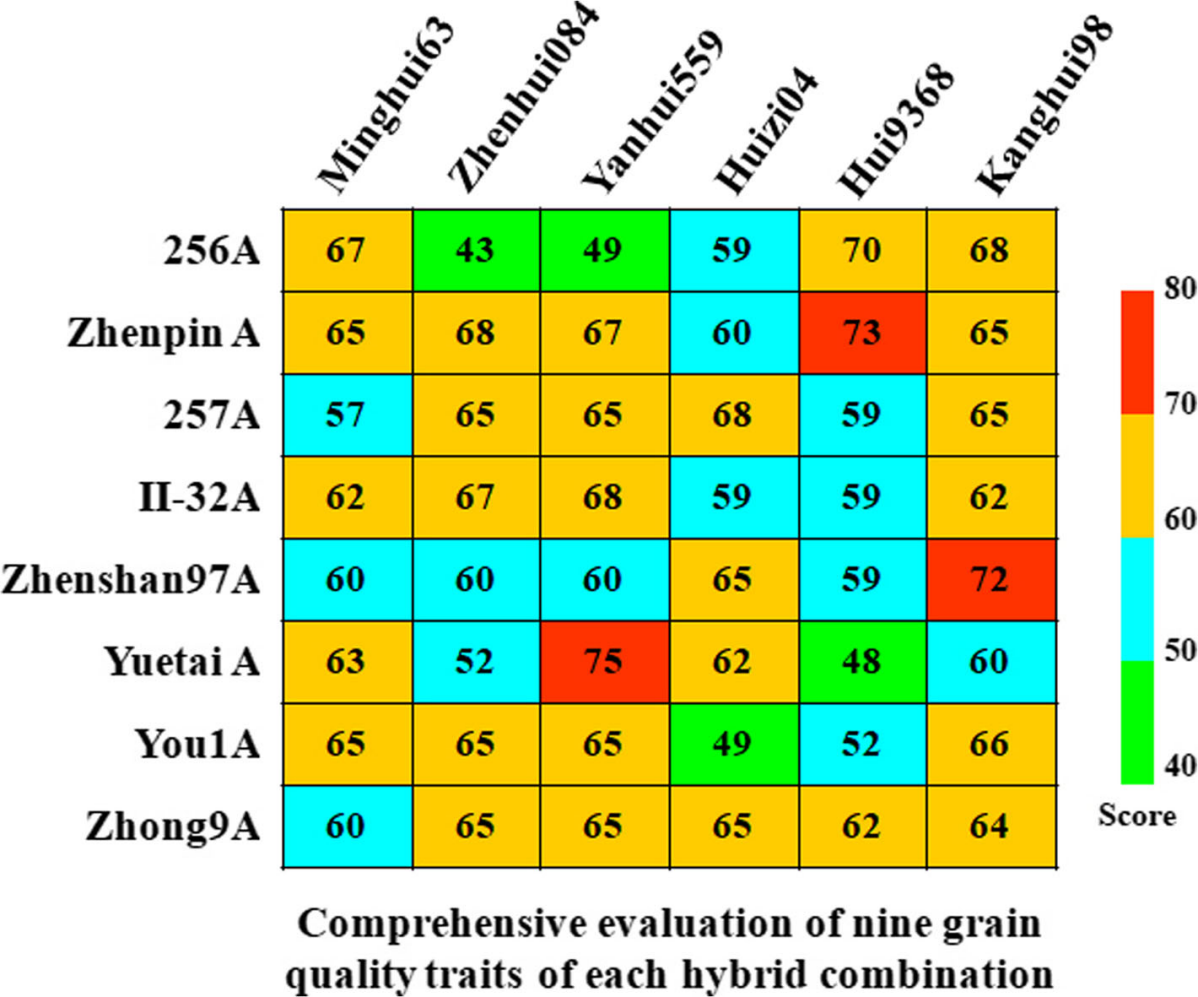
Comprehensive evaluation scores of nine grain quality traits of 48 F_2_s of Indica rice

**Fig. 2:**
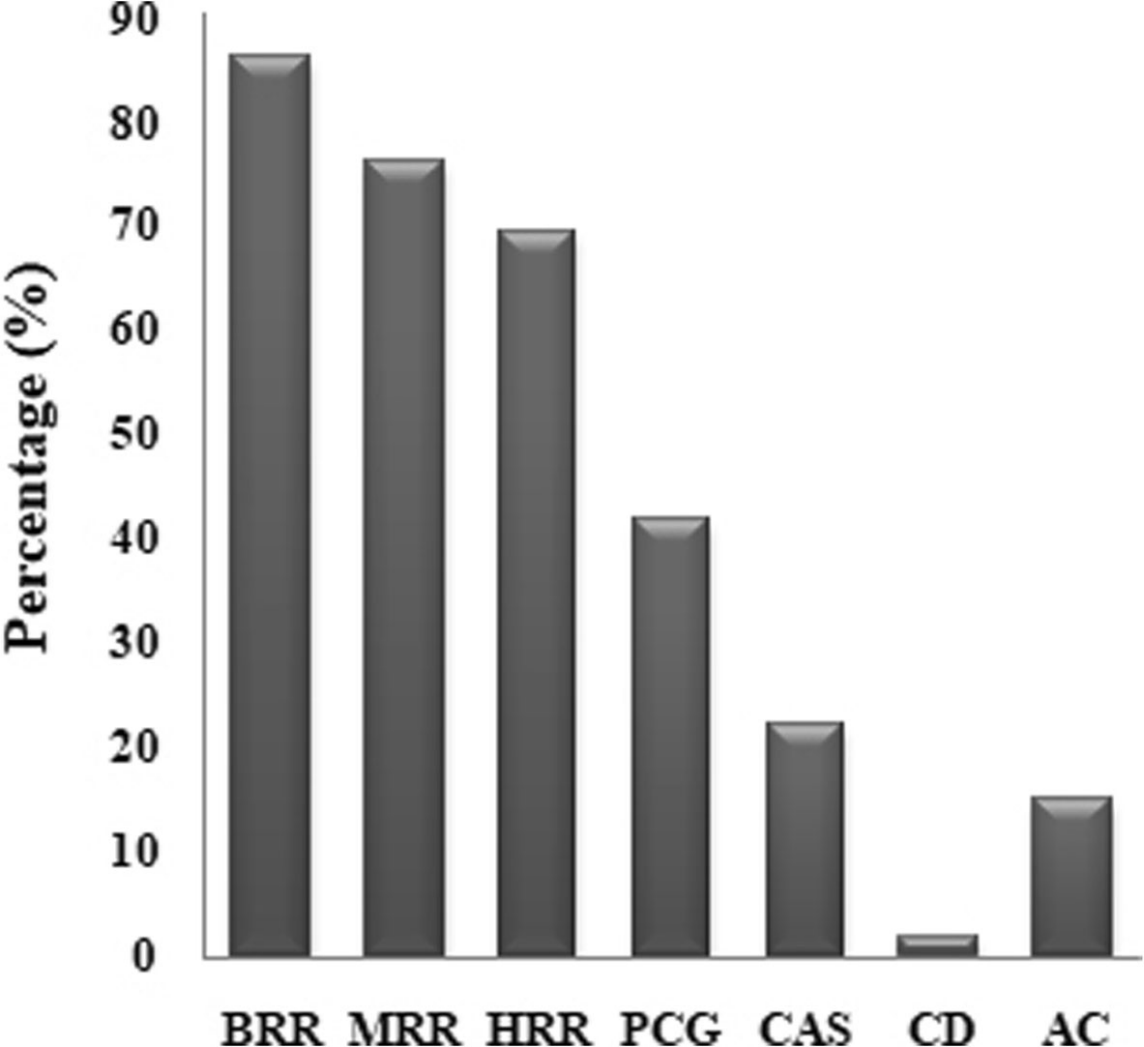
The values of BRR, MRR, HRR, PCG, CAS, CD and AC of F_2_ crossed by Yuetai A and Yanhui559

Based on the five level evaluation criteria and comprehensive scoring standards for nine grain quality traits in indica rice shown in Additional file 3: Table S3. the comprehensive evaluation scores of 48 F_2_s ranged from 43 to 75 (Full score of nine traits is 90) (Fig. 1). The combination crossed by Yuetai A and Yanhui559 recorded the highest score among the 48 F_2_s (Fig. 1). The grain quality performance of F_2_ derived from the combination of Yuetai A × Yanhui559 were showed in Fig. 2. According to China’s Ministry of Agriculture’s Edible Rice Quality Industry Standard (NY/T 593-2002) [29], the quality traits of grain could be divided into five levels and the first level is the best. The HRR, MRR, BRR and CD of F_2_ grains crossed by Yuetai A and Yanhui559 belonged to level 1. The GT and AC belonged to level 2. And the remaining traits, i.e. CAS, PCG and GC belonged to level 3, 3 and 4, respectively (Fig. 2). The comprehensive evaluation scores of the combinations considering Yuetai A, Zhenpin A, Zhenshan97A, Yanhui559, Hui9368 and Kanghui98 as parents were generally higher, which was basically consistent with the results of general combining ability analysis (Fig. 1 and Table 1).

### Estimations of GCA effects of japonica CMS and restorer lines

The parents of japonica hybrid rice showed both positive and negative GCA effects values for 9 quality-related traits. Among the 13 japonica CMS lines, CMS 863 A was observed to be the best general combiner for all the studied traits except GT (Table 2). Maximum GCA value of gelatinization temperature was showed by 6427 A.

**Table 2:** Effect values of GCA of Japonica CMS and restorer lines for 9 quality-related traits

CMS lines	BRR (%)	MRR (%)	HRR (%)	PCG (%)	CAS (%)	CD (%)	GT (ASS)	GC mm)	AC (%)
863A	3.5a	3.1a	2.8a	2.9a	2.6a	−0.06 L	0.44b	2.9a	2.0a
9201A	−2.5 fg	−2.2 g	−2.0f	−2.0 g	−1.8 g	0.03d	−0.59j	− 2.1 fg	− 1.6i
Xu 2A	1.8abc	1.6bc	1.4b	1.5c	1.3c	−0.05 k	0.40c	1.6bc	1.2c
Nanjing 46A	−0.4cde	0.4de	0.3d	0.2de	0.2de	−0.03 h	0.13f	0.4cde	0.3e
731A	−0.9ef	−0.8f	− 0.6e	−0.5f	− 0.4f	0.08b	0.04 h	−0.9ef	0.0 g
Liuqianxin A	−4.2 g	−3.7 h	−3.4 g	−3.4 h	−3.0 h	0.06c	−0.89 L	−3.6 h	−2.9 k
6427A	2.9ab	2.6ab	2.3a	2.3b	2.0b	−0.06 m	0.48a	2.6ab	1.7b
Zhendao 88A	−3.9 g	−3.4 h	−3.1 g	− 3.0 h	−2.7 h	0.10a	−0.70 k	− 3.3gh	− 2.2j
Qingkong A	−0.3 cde	0.2ef	0.2d	0.2de	0.2de	−0.02 g	0.02i	0.1de	0.2f
Yueguang A	0.8 cde	0.7cde	0.6 cd	0.5d	0.4d	−0.04j	0.11 g	0.8 cd	0.6d
Wuqiang A	1.5bcd	1.3 cd	1.2bc	1.1c	1.0c	−0.03i	0.29d	1.3 cd	0.7d
Wuyujing 3A	0.0de	0.0ef	0.0de	−0.1ef	−0.1e	− 0.01f	0.11 g	0.1de	−0.6 h
Liuyan 189A	0.3 cde	0.3de	0.3d	0.3de	0.3d	0.03e	0.18e	0.2de	0.6d
Restorers									
C418	2.2a	2.8a	3.5a	3.6a	3.7a	−0.12f	0.63a	2.8a	3.1a
Ninghui8hao	1.4a	1.3b	1.5b	1.5b	1.4b	−0.04d	0.29b	1.3b	1.3b
Yunhui 4 hao	0.6a	0.7bc	1.2bc	1.1b	1.1b	−0.05e	0.05d	0.8b	1.0c
Zhehui 315	1.3a	0.5bc	0.1d	0.1c	−0.3d	0.01b	−0.03e	0.4b	−0.1e
Yanhui R50	−5.6c	−5.6d	−6.8e	−6.8d	−6.4e	0.24a	−1.05f	−5.7c	−5.5f
Xiushui 04R	0.1b	0.2c	0.5 cd	0.4c	0.4c	−0.03c	0.11c	0.3b	0.3d

The Japonica CMS and restorer lines trail by alphabets are significantly different at *P* < 0.01

Among the 6 japonica rice restorer lines, C418 recorded the maximum GCA effects for most of traits; Ninghui8hao, Yunhui4hao also showed good general combiners for all the studied traits (Table 2). In terms of GCA performances of all quality-related traits, CMS 863 A, 6427 A and restorers C418, Ninghui8hao and Yunhui4hao had a favorable GCA effects for developing japonica hybrids of superior performances.

**Fig. 3:**
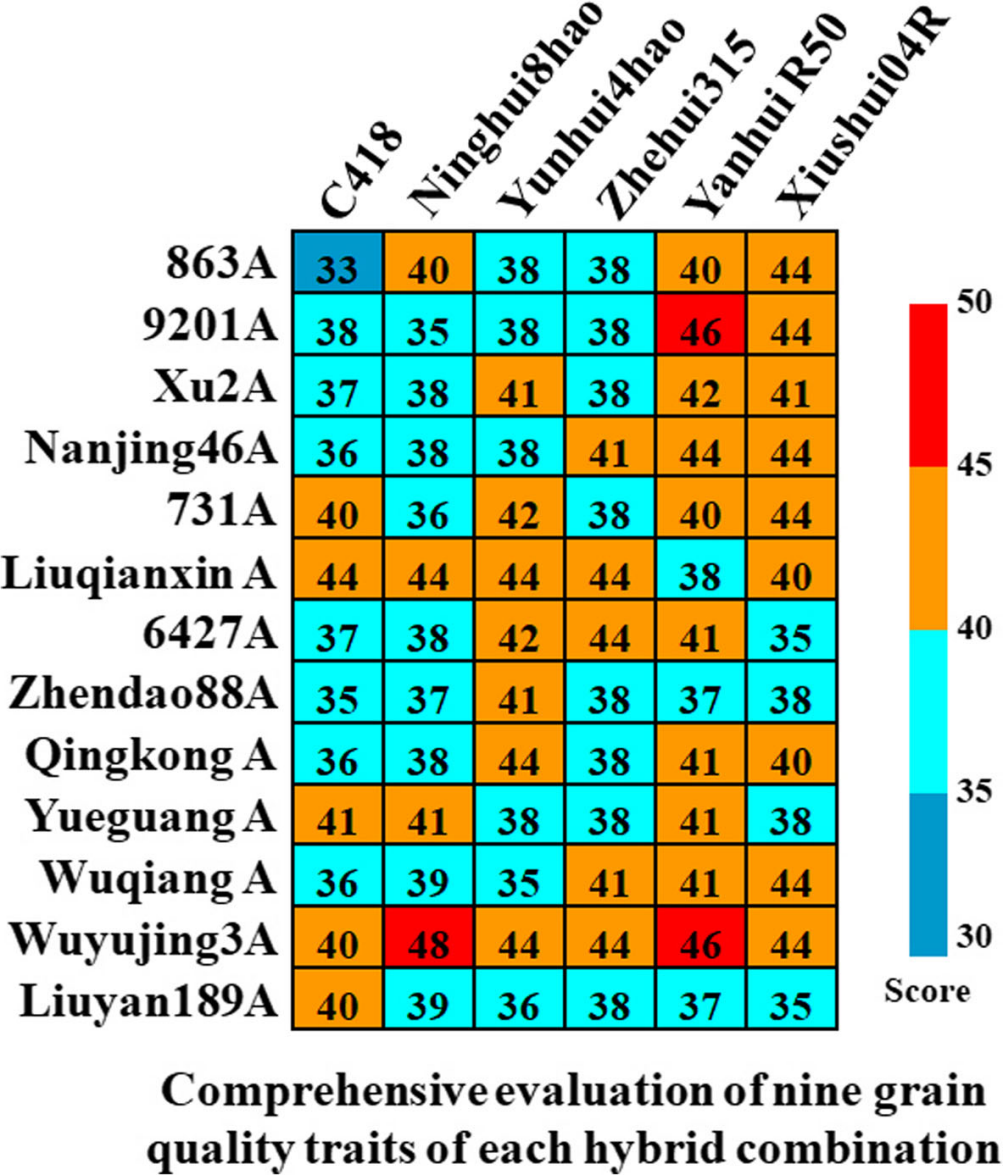
Comprehensive evaluation scores of nine grain quality traits of 78 F_2_s of Japonica rice

**Fig. 4:**
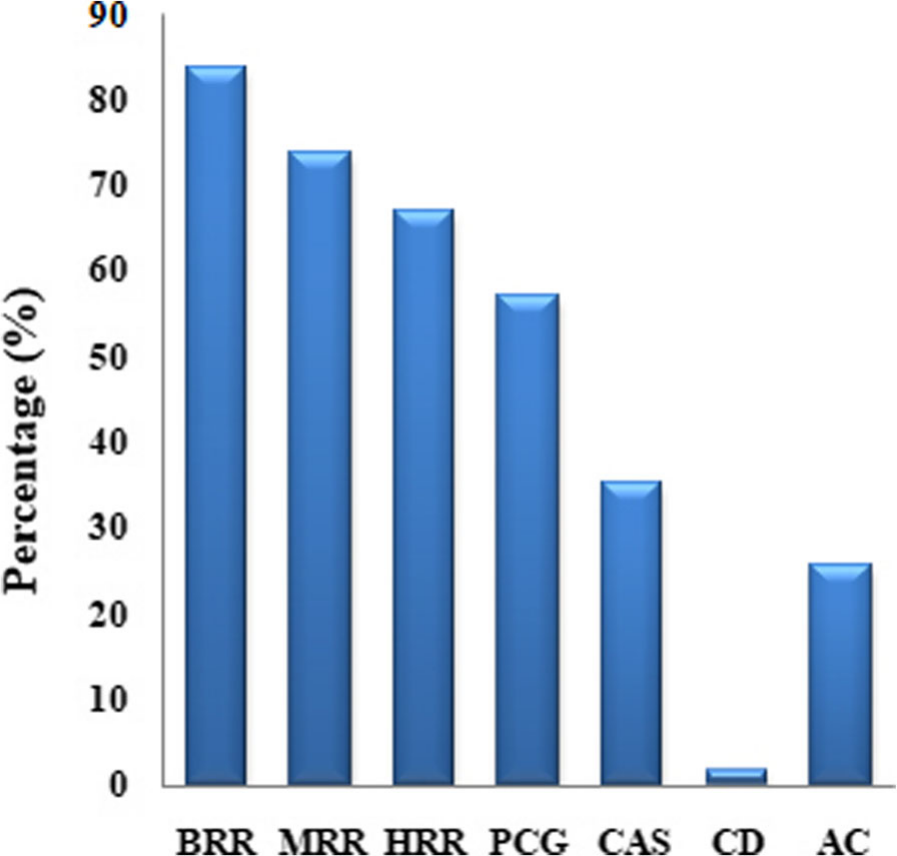
The values of BRR, MRR, HRR, PCG, CAS, CD and AC of F_2_ crossed by Wuyujing3A and Ninghui8hao

Based on the five level evaluation criteria and comprehensive scoring standards for the nine grain quality traits shown in Additional file 4: Table S4. the comprehensive evaluation scores of 78 F_2_s ranged from 33 to 48 (Full score of nine traits is 90) (Fig. 3). The highest score was observed in the combination crossed by Wuyujing3A and Ninghui8hao (Fig. 3). Figure 4 showed the values of 7 grain quality traits of the aforementioned cross. According to the NY/T 593-2002 mentioned above, the CD and BRR of F_2_ grains in the combination crossed by Wuyujing3A and Ninghui8hao belonged to level 2; the HRR, MRR, GT and GC belonged to level 3; and the remaining traits, i.e. PCG, CAS and AC belonged to level 4, 5 and 5, respectively (Fig. 4). The comprehensive evaluation scores of the combinations considering Wuyujing3A, 92101A, Ninghui8hao and Yanhui R50 as parents were generally higher, which was basically consistent with the results of general combining ability analysis (Fig. 3 and Table 2).

### Association analysis between constructed SNPLDBs and GCA effects

**Fig. 5:**
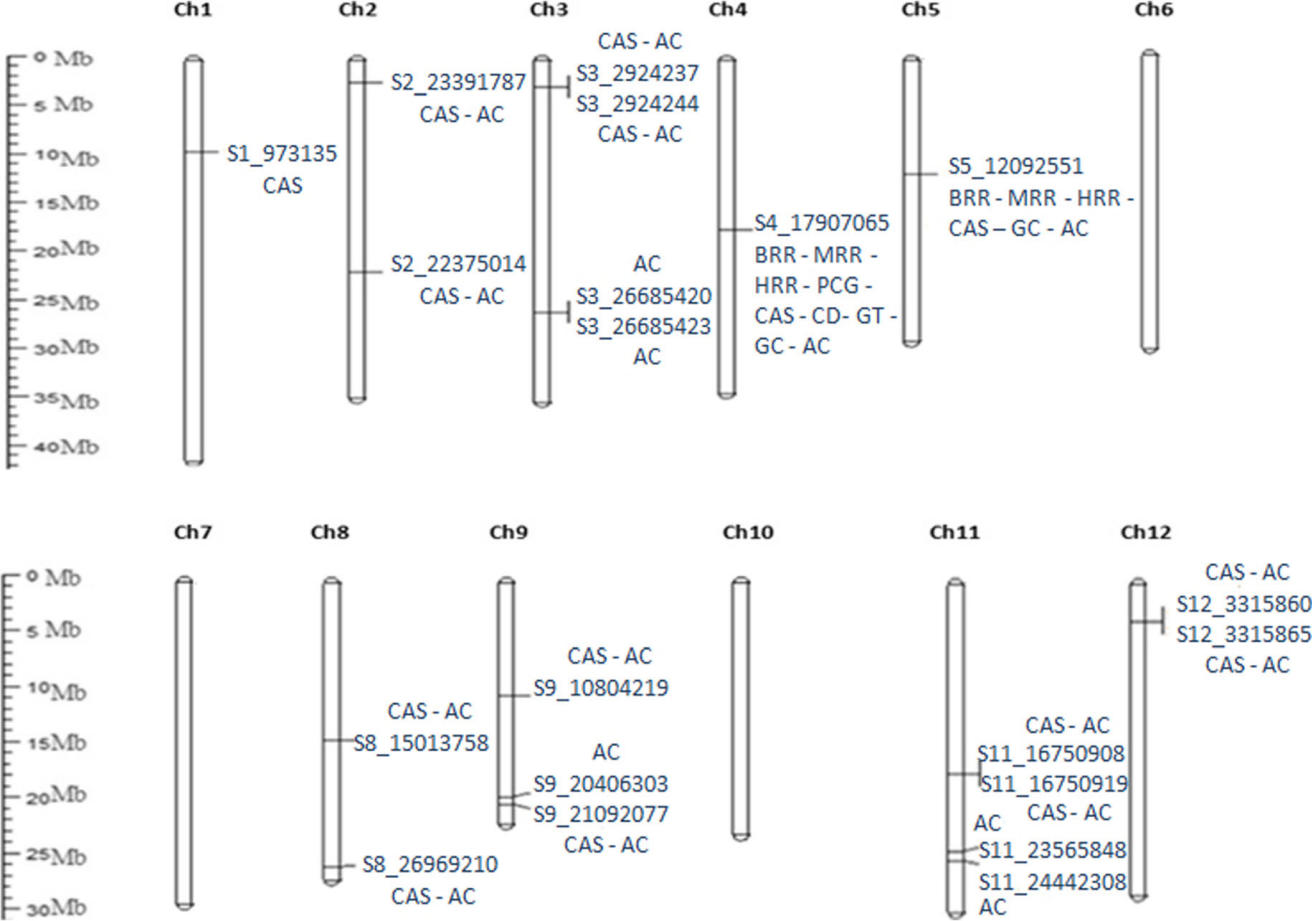
SNPLDBs positions on chromosomes associated with the GCA of traits. BRR, brown rice rate; MRR, milled rice rate; HRR, head rice rate; PCG, percentage of chalky grains; CAS, chalky area size; CD, chalkiness degree; GT, Gelatinization Temperature; GC, Gel Consistency; AC, Amylose Content

The association analysis between the effect values of GCA and constructed SNPLDBs revealed a total of 39 significant SNPLDBs for GCA of 9 quality-related traits. The identified SNPLDBs were distributed on nine of the 12 chromosomes of rice. The number of associated SNPLBDs for each trait varied and, on average over the 39 SNPLDBs, 41.6% of phenotypic variation was explained by each SNPLDB. The detail information of the 39 associated SNPLDBs is presented in Fig. 5 and Table 3.

**Table 3:** List of significant SNPLDBs associated with GCA effects of 9 Quality-related traits

Trait	SNPLBDs	Chromosome	*P*-value	*R*^*2*^(%)
BR	4_BLOCK_17882078_17907416	4	0.00004	54.9
	S5_12092551	5	0.00083	49.1
MR	4_BLOCK_17882078_17907416	4	0.00007	53.6
	S5_12092551	5	0.00078	49.4
HR	4_BLOCK_17882078_17907416	4	0.00051	52.0
	S5_12092551	5	0.00073	49.9
	8_BLOCK_26862470_27057202	8	0.00080	35.5
PCG	4_BLOCK_17882078_17907416	4	0.00093	51.1
CAS*	1_BLOCK_937039_1124378	1	0.00352	34.3
	S2_22375014	2	0.00277	29.4
	2_BLOCK_23246549_23402926	2	0.00978	19.6
	3_BLOCK_2736967_2935082	3	0.00679	33.6
	4_BLOCK_17882078_17907416	4	0.00135	59.0
	S5_12092551	5	0.00226	43.1
	S8_15013758	8	0.00232	45.6
	8_BLOCK_26862470_27057202	8	0.00483	42.9
	S9_10804219	9	0.00431	47.8
	S9_21092077	9	0.00520	26.4
	11_BLOCK_16710912_16770852	11	0.00319	60.9
	12_BLOCK_3214955_3413848	12	0.00702	23.2
CD	4_BLOCK_17882078_17907416	4	0.00075	52.2
GT	4_BLOCK_17882078_17907416	4	0.00009	50.3
GC	4_BLOCK_17882078_17907416	4	0.00009	50.3
	S5_12092551	5	0.00057	41.3
AC*	S2_22375014	2	0.00343	47.8
	2_BLOCK_23246549_23402926	2	0.00902	20.0
	3_BLOCK_2736967_2935082	3	0.00909	51.0
	3_BLOCK_26597888_26785589	3	0.00879	23.6
	4_BLOCK_17882078_17907416	4	0.00281	54.3
	S5_12092551	5	0.00235	42.9
	S8_15013758	8	0.00124	49.5
	8_BLOCK_26862470_27057202	8	0.00707	49.8
	S9_10804219	9	0.00731	43.7
	9_BLOCK_20278565_20464993	9	0.00954	19.8
	S9_21092077	9	0.00533	26.2
	11_BLOCK_16710912_16770852	11	0.00164	63.5
	11_BLOCK_23565814_23565855	11	0.00973	20.9
	11_BLOCK_24442254_24636131	11	0.00759	31.9
	12_BLOCK_3214955_3413848	12	0.00791	22.6

^*^(max *P* value 0.01)

### Brown rice rate

Two SNPLDBs situated on 2 different chromosomes (Chr4, Chr5) showed significant associations with the GCA of brown rice rate. The associated GCA-SNPLDBs of brown rice rate explained phenotypic variance in the range of 49.1% (S5_12092551) to 54.9% (4_BLOCK_17882078_17907416) (Table 3). The SNPLDB detected on chromosome 4 showed a positive effect with GCA of the trait. The elite SNP genotype (A/C at 17907065 bp position) of gene Os04g0368800/LOC_Os04g30010 situated on chromosome 4 increased BRR by 10.28% (Table 4).

**Table 4:** Annotations of detected genes within the intervals of associated SNPLDBs of GCA of 9 quality related traits

Traits	Associated SNPLBDs	Elite SNP genotypes	Chr	position (bp)	Associated gene	Average of homozygous group(1)	Average of heterozygous group(2)	Increments (%) [(2)–(1)]/(1)
BRR	4_BLOCK_17882078_17907416	A/C	4	17,907,065	Os04g0368800	68.59	75.64	10.28
	S5_12092551	A/T	5	12,092,551				
MRR	4_BLOCK_17882078_17907416	A/C	4	17,907,065	Os04g0368800	59.90	66.26	10.62
	S5_12092551	A/T	5	12,092,551				
HRR	4_BLOCK_17882078_17907416	A/C	4	17,907,065	Os04g0368800	53.40	60.26	12.85
	S5_12092551	A/T	5	12,092,551				
	8_BLOCK_26862470_27057202	C/T	8	26,969,210	Os08g0539400	51.56	60.50	17.35
PCG	4_BLOCK_17882078_17907416	A/C	4	17,907,065	Os04g0368800	48.63	43.46	−10.63
CAS	1_BLOCK_937039_1124378	A/G	1	973,135	Os01g0117200	29.00	27.58	−4.89
	S2_22375014	C/T	2	22,375,014				
	2_BLOCK_23246549_23402926	A/C	2	23,391,787	Os02g0599200	27.75	25.65	−7.55
	3_BLOCK_2736967_2935082	C/T	3	2,924,237	Os03g0153000	28.80	27.58	−2.81
		A/G		2,924,244	Os03g0153000	28.80	27.58	−2.81
	4_BLOCK_17882078_17907416	A/C	4	17,907,065	Os04g0368800	27.85	22.95	−17.58
	S5_12092551	A/T	5	12,092,551				
	S8_15013758	G/T	8	15,013,758				
	8_BLOCK_26862470_27057202	C/T	8	26,969,210	Os08g0539400	27.28	21.09	−22.68
	S9_10804219	A/C	9	10,804,219	Os09g0345600	28.13	27.03	−3.90
	S9_21092077	A/C	9	21,092,077				
	11_BLOCK_16710912_16770852	A/C	11	16,750,908	Os11g0479100	28.94	27.67	−4.37
		A/T		16,750,919	Os11g0479100	28.94	27.67	−4.37
	12_BLOCK_3214955_3413848	A/C	12	3,315,860	Os12g0165000	27.37	23.71	−13.38
		G/T		3,315,865	Os12g0165000	27.37	23.71	−13.38
CD	4_BLOCK_17882078_17907416	A/C	4	17,907,065	Os04g0368800	1.96	1.76	−10.47
GT	4_BLOCK_17882078_17907416	A/C	4	17,907,065	Os04g0368800	2.83	4.06	43.57
GC	4_BLOCK_17882078_17907416	A/C	4	17,907,065	Os04g0368800	47.91	52.17	8.90
	S5_12092551	A/T	5	12,092,551				
AC	S2_22375014	C/T	2	22,375,014				
	2_BLOCK_23246549_23402926	A/C	2	23,391,787	Os02g0599200	20.49	18.60	−9.25
	3_BLOCK_2736967_2935082	C/T	3	2,924,237	Os03g0153000	21.15	20.30	−3.99
		A/G		2,924,244	Os03g0153000	21.15	20.30	−3.99
	3_BLOCK_26597888_26785589	A/G	3	26,685,420				
		A/G		26,685,423				
	4_BLOCK_17882078_17907416	A/C	4	17,907,065	Os04g0368800	20.62	16.38	−20.57
	S5_12092551	A/T	5	12,092,551				
	S8_15013758	G/T	8	15,013,758				
	8_BLOCK_26862470_27057202	C/T	8	26,969,210	Os08g0539400	20.09	14.73	−26.65
	S9_10804219	A/C	9	10,804,219	Os09g0345600	20.86	19.89	−4.67
	9_BLOCK_20278565_20464993	C/G	9	20,406,303				
	S9_21092077	A/C	9	21,092,077				
	11_BLOCK_16710912_16770852	A/C	11	16,750,908	Os11g0479100	21.89	20.58	−5.97
		A/T		16,750,919	Os11g0479100	21.89	20.58	−5.97
	11_BLOCK_23565814_23565855	A/T	11	23,565,848	Os11g0609700	20.16	17.69	−12.23
	11_BLOCK_24442254_24636131	A/C	11	24,442,308				
	12_BLOCK_3214955_3413848	A/C	12	3,315,860	Os12g0165000	20.14	16.91	−16.04
		G/T		3,315,865	Os12g0165000	20.14	16.91	−16.04

### Milled rice rate

Two SNPLDBs situated on 2 different chromosomes (Chr4, Chr5) showed significant relationships with the GCA of milled rice rate. The phenotypic variation caused by these SNPLDBs ranged from 49.4% (S5_12092551) to 53.6% (4_BLOCK_17882078_17907416) (Table 3). The SNPLDB detected on chromosome 4 showed a positive effect on GCA of MRR. The elite SNP genotype (A/C at 17907065 bp position) of gene Os04g0368800/LOC_Os04g30010 situated on chromosome 4 increased MRR by 10.62% (Table 4).

### Head rice rate

Three SNPLDBs distributed over chromosome 4, 5, and 8 revealed significant associations with the GCA of head rice rate. The phenotypic variations caused by these associated SNPLDBs were 52.0% (4_BLOCK_17882078_17907416), 49.9% (S5_12092551) and 35.5% (8_BLOCK_26862470_27057202), respectively (Table 3). The SNPLDB (4_BLOCK_17882078_17907416) detected on chromosome 4 favored larger phenotypic variation and both SNPLDBs on chromosomes 4 and 8 showed a positive effect on the GCA of HRR. The elite SNP genotypes (AC/CT at the position of 17,907,065 bp and 26,969,210 bp) of genes Os04g0368800 and Os08g0539400 located on chromosomes 4 and 8 increased HRR by 12.85 and 17.35%, respectively (Table 4).

### Percentage of chalky grains

One SNPLDB situated on chromosome 4 showed associations with the GCA of the percentage of chalky grains. The phenotypic variance explained by the SNPLDB (4_BLOCK_17882078_17907416) was 51.1% (Table 3). The SNP genotype (A/C at 17907065 bp position) of gene Os04g0368800 situated on chromosome 4 decreased PCG by 10.63% (Table 4).

### Chalky area size

Twelve SNPLDBs situated on 9 various chromosomes (Chr1, Chr2, Chr3, Chr4, Chr5, Chr8, Chr9, Chr11 and Chr12) were associated with the GCA of chalky area size. The percentages of phenotypic variation explained by these SNPLDBs were ranged from 19.6% (2_BLOCK_23246549_23402926) to 60.9% (11_BLOCK_16710912_16770852) (Table 3). Among the eight genes associated with the combining ability of CAS, the SNP genotype of gene Os08g0539400 (C/T at 26969210 bp position) situated on chromosome 8 recorded the largest decrement 22.68% (Table 4).

### Chalkiness degree

One SNPLDB situated on chromosome 4 showed associations with the GCA of chalkiness degree. The phenotypic variance caused by the SNPLDB was 52.2% (4_BLOCK_17882078_17907416) (Table 3). The elite SNP genotype (A/C at 17907065 bp position) of gene Os04g0368800 situated on chromosome 4 made CD of heterozygous group decreased from 1.96 to 1.76% (Table 4).

### Gelatinization temperature

One SNPLDB situated on chromosome 4 showed associations with the GCA of gelatinization temperature. The phenotypic variance caused by the SNPLDB (4_BLOCK_17882078_17907416) was 50.3% (Table 3). The SNP genotype (A/C at 17907065 bp position) of gene Os04g0368800 situated on chromosome 4 in the heterozygous group has a 43.57% larger GT than that in homozygous group (Table 4).

### Gel consistency

Two SNPLDBs situated on 2 different chromosomes (Chr4, Chr5) revealed significant relationships with the GCA of gel consistency. The percentage of phenotypic variation explained by these SNPLDBs ranged from 41.3% (S5_12092551) to 50.3% (4_BLOCK_17882078_17907416) (Table 3) The elite SNP genotype (A/C at 17907065 bp position) of gene Os04g0368800 situated on chromosome 4 increased GC by 8.9% (Table 4).

### Amylose content

Fifteen SNPLDBs situated on 8 various chromosomes (Chr2, Chr3, Chr4, Chr5, Chr8, Chr9, Chr11 and Chr12) were found associated with the GCA of amylose content. Three significant SNPLDBs were observed on chromosome 9 and 11, respectively. The percentage of phenotypic variations explained by these SNPLDBs ranged from 19.8% (9_BLOCK_20278565_20464993) to 63.5% (11_BLOCK_16710912_16770852) (Table 3). A total of eight SNPLDBs, 11 elite SNPs genotype were detected decreasing GCA of AC. The highest decrease percentage between the heterozygous group and the homozygous group is the elite SNP genotype (C/T at 26969210 bp position) ofOs08g0539400 situated on chromosome 8, and the decreased value was 26.65% (Table 4).

### Distribution of superior alleles among the 33 parents

In this study, the alleles which caused positive effects on GCA of quality traits of parents of hybrid rice were considered as the superior alleles. For BRR, MRR, HRR and GC traits, the higher GCA effect value was considered as positive effects, and for PCG, CAS, CD, GT and AC traits, the lower GCA effect value was considered positive effects. The number of superior alleles for BRR, MRR, HRR, PCG, CAS, CD, GT, GC and AC detected across 33 parents were 4, 4, 16, 2, 66, 2, 2, 4 and 90, respectively (Table 5).

**Table 5:** Distribution of superior GCA alleles for the 9 quality related traits among the 33 parents used in this study

Parents	BR	MR	HR	PCG	CAS	CD	GT	GC	AC
Indica CMS line 256A	–	–	1	–	2	–	–	–	3
Zhenpin A	–	–	1	–	4	–	–	–	7
257A	–	–	1	–	3	–	–	–	3
II-32A	–	–	1	–	1	–	–	–	1
Zhenshan 97A	–	–	1	–	4	–	–	–	4
Yuetai A	–	–	1	–	4	–	–	–	6
You 1A	–	–	1	–	1	–	–	–	1
Zhong 9A	–	–	–	–	3	–	–	–	3
Indica Restorer									
Minghui 63	–	–	1	–	6	–	–	–	8
Zhenhui 084	1	1	1	–	5	–	–	1	7
Yanhui 559	–	–	1	–	2	–	–	–	2
Huizi 04	–	–	–	–	1	–	–	–	1
Hui 9368	–	–	–	–	2	–	–	–	4
Kanghui98	–	–	–	–	2	–	–	–	5
Japonica CMS line									
863A	–	–	–	–	1	–	–	–	–
9201A	–	–	–	–	2	–	–	–	2
Xu 2A	–	–	–	–	–	–	–	–	–
Nanjing 46A	–	–	–	–	1	–	–	–	1
731A	–	–	–	–	–	–	–	–	–
Liuqianxin A	–	–	–	–	2	–	–	–	2
6427A	–	–	–	–	–	–	–	–	–
Zhendao 88A	1	1	1	1	2	1	1	1	2
Qingkong A	–	–	–	–	1	–	–	–	1
Yueguang A	–	–	–	–	–	–	–	–	–
Wuqiang A	–	–	–	–	2	–	–	–	2
Wuyujing 3A	–	–	–	–	1	–	–	–	1
Liuyan 189A	–	–	–	–	–	–	–	–	–
Japonica Restorer									
C418	–	–	1	–	1	–	–	–	3
Ninghui8hao	–	–	–	–	4	–	–	–	7
Yunhi 4 hao	–	–	1	–	1	–	–	–	1
Zhehui 315	–	–	1	–	2	–	–	–	4
Yanhui R50	2	2	2	1	6	1	1	2	9
Xiushui 04R	–	–	–	–	–	–	–	–	–

Among the 8 Indica CMS lines, CMS Zhenpin A showed 12 positive GCA alleles, including one for HRR, 4 for CAS and 7 for AC.. CMS Yuetai A demonstrated 11 positive GCA alleles, including one for HRR, 4 for CAS and 6 for AC (Table 5). Meanwhile, among the 6 Indica restorer lines, restorer Minghui 63 had 15 positive GCA alleles, including one for HRR, 6 for CAS and 8 for AC. Restorer Zhenhui 084 contained 16 positive GCA alleles, including one for BRR, 1 for MRR, 1 for HRR, 5 for CAS, 1 for GC and 7 for AC.. Restorer Kanghui98 contained 2 positive GCA alleles for CAS and 5 positive GCA alleles for AC.

Among the 13 Japonica CMS lines, CMS Zhendao 88A had a maximum number of positive GCA alleles for all traits. The CMS 9201A, Liuqianxin A and Wuqiang A each had 2 positive GCA alleles for CAS and AC, respectively (Table 5). Among the 6 Japonica restorer lines, restorer Yanhui R50 had 26 GCA alleles in genomes for all the 9 quality. The restorer Ninghui8hao showed the presence of 4 and 7 positive GCA alleles for CAS and AC, respectively. The restorer Zhehui315 carried 1, 2 and 4 positive GCA alleles for HRR, CAS and AC, respectively. The restorer C418 contained 1,1 and 3 positive GCA alleles for HRR, CAS and AC, respectively.

### Elite parental lines for developing of superior hybrid cultivars

Based on the presence of detected superior GCA-SNPLDB alleles that could be combined (pyramided) into a maintainer (CMS has no fertile pollens) or restorer lines, the elite parental combinations of improved GCA of quality-related traits were suggested. The superior alleles carried by the CMS and restorer lines are presented in (Table 5). The parental lines carrying a maximum number of superior GCA-SNPLDB alleles could be the elite parental lines of superior GCA performances, which had the potential of developing hybrids of promising performances. Alleles detected in CMS and restorer lines will improve the GCA of traits which will alternatively cause positive effect on rice quality trait values of F_2_ bulk population.

Among the Indica CMS and restorer lines, the crossing combinations between CMS Zhenpin A and restorers Minghui 63 and Kanghui98 are the elite Indica parental lines for developing of superior hybrids of head rice rate, chalky area size and amylose content. Subsequently, among the Japonica parents of hybrid rice, CMS 863A, Liuqianxin A, Wuqiang A and Zhendao 88A with restorers C418, Yanhui R50 and Ninghui8hao were found to be elite parental combinations containing a maximum number of superior GCA alleles for most of the quality-related traits.

## Discussion

In this study, we prepared two sets of F_1_ crosses using NCII genetic design to evaluate GCA effect values of 9 quality-related traits in parents in rice. One set was consisted of 48 F_1_ crosses made by 8 CMS and 6 restorer lines within indica rice subspecies, and the other set contained 78 F_1_ crosses made by 13 CMS and 6 restorer lines within japonica rice subspecies. Significant variations of the GCA effect values of the 9 traits were observed in 33 parents. Among the parental lines in Indica subspecies, CMS Zhenpin A, Zhenshan97 A, and 257A and restorers Kanghui98, Minghui63 and Yanhui559 were recognized as good general combiners due to their maximum positive GCA effect values for most of the traits. Among the parental lines in Japonica subspecies, CMS 863A, 6427A and Xu 2A and restorers C418, Ninghui8hao and Yunhui4hao recorded maximum positive GCA effect values for traits.

By association analysis between the effects of GCA and SNPLDBs, we detected 39 significant SNPLDB loci associated with the GCA of the 9 traits, and the SNPLDB loci were located on chromosome 1 (1), 2 (4), 3 (3), 4 (9), 5 (6), 8 (5), 9 (5), 11 (4) and 12 (2). By searching the website www.ricedata.cn/gene/index.htm, we found that on chromosome 2, our associate region 23,246,549 − 23,402,926 responsible for GCA of AC/CAS was near to the cloned rice quality-related gene *du3* (*OsCBP20*, *Os02g0612300*, 24,100,174 − 24,103,481), away from 697,248 bp (24,100,174 − 23,402,926) each other. Also, on chromosome 9, the associate region (S9_21,092,077) responsible for GCA of CAS/AC was 343,568 bp apart from the cloned rice quality-related gene *OsVPS*22 (*Os09g0529700*, 20,744,838 − 20,748,509). We did not find SNPLDB loci harboring the cloned *Wx* and *ALK* genes on chromosome 6 associated with GCA of the these traits. Togather, we inferred that the genetic basis of trait GCA in the parents is different from that of trait itself in rice. And we explained the results as that trait GCA in the parents is mainly related with polygenes rather than with major gene controlling the trait itself.

From the perspective of enhancing the hybrid rice quality, F_2_ bulk seed analysis is effective and unavoidable choice in our study, and it is feasible to associated SNPLDB with combining ability for grain quality traits in hybrid rice parents through combining the SNP genotypes of parents and the quality phenotypes of F_2_ directly. All the elite SNPLDBs detected in our research are useful for enhancement grain quality traits in hybrid rice.

### Significance of GCA evaluation by markers

Traditional methods, such as Grifing’s mehods, NC methods, can be used to estimate the GCA effects of parental lines. However, if we want to improve GCA of parents, we need to breed new parents by many years and evaluate them again. It is a time-consuming and laborious work in field trials. The ongoing progress in genomics has provided a wide range of molecular markers for crop improvement. Since there are enormous types of molecular markers are available for tagging the genomic regions of important phenotypes in parental genomes of hybrid rice, the information of GCA may become predictable based on dissection of parental lines using markers. By genotyping GCA of parents with the use of molecular markers, we can not only detect the GCA loci in parental genomes but can also improve the GCA of a parent by pyramiding the favorable GCA alleles exiting in various parents into a single parent (maintainer (CMS) or restorer) and/or removing of unfavorable GCA alleles by marker-assisted selection. Previously, based on the identified favorable and unfavorable SSR alleles, the combining ability of an elite rice restorer line (Minhui63) was enhanced by the incorporation of favorable combining ability alleles and elimination of unfavorable CA alleles [21].

### Advantage of SNPLDBs for association analysis

SNPs always show more biological significance when SNP clusters are tightly organized into haplotype blocks in the examined genomic region [28]. Association analysis with SNPLDBs increases the power of association [6, 30]. In fact, compared with individual SNPs, constructed SNPLDB has a higher meaning, enhancing the accuracy and robustness of association analysis [8, 11]. Association analysis using SNPLDB can significantly improve the efficiency of detecting QTLs [23, 24, 38]. In addition, association analysis using SNPLDB can provide new biological insights for genomic regions that determine trait control, which is not available with a single SNP method [1]. Efforts to construct SNPLDB from large genomic sequence data are being successful in various crops, such as Chinese soybean [40], rice [15], and corn [36], and wheat [9].

### Association analysis by single-factor test

To date, many studies were conducted to dissect the genomic regions (or QTLs) underlying important phenotypes of rice using marker-trait association strategy. Different methods were used for different populations. GWAS method is suitable for natural population. Interval mapping method is suitable for bi-parent-derived segregation populations. For our NCII-derived population, the single-factor test method was used for testing associations between marker and phenotype [26]. This association method provides maximum resolution that enables the identification of significant candidate loci. Using this single-factor association analysis, we found 39 significant SNPLDBs associated with the GCA of 9 quality-related traits. The SNPLDBs distributed on chromosome 1, 2, 3, 4, 5, 8, 9, 11 and 12 (Table 3). According to the distribution of the superior SNPLDB alleles (Table 5), GCA of the quality traits in parents could be improved by pyramiding or substituting process though molecular marker assisted selection. For example, GCA of the 9 traits in japonica restorer Xiushui 04R could be enhanced by crossing it with another japonica restorer Yanhui R50 (Table 5).

## Conclusions

We identified 39 significant SNPLDBs associated with the GCA of 9 quality-related traits among the parents used and the SNPLDB information could be used to improve the GCA of parents for the quality traits in the future. The genetic basis of trait GCA in parents is different from that of trait itself.

## Methods

### Field experiment

**Fig. 6:**
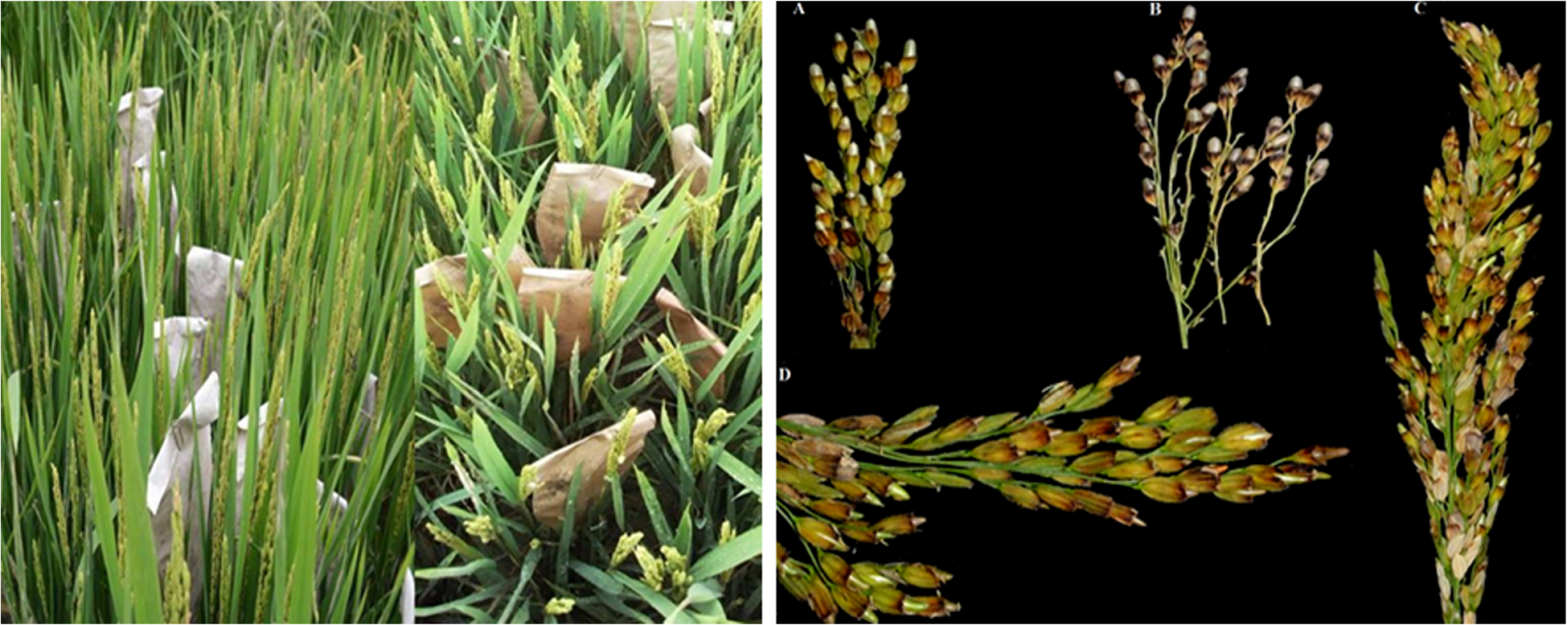
Paper bags covering panicles of CMS plants pollinated by the restorer pollens in paddy field (left) and the resultant seed-set panicles (right). A and B are the japonica CMS panicles containing the japonica F_1_ seeds; C and D are the indica CMS panicles containing the indica F_1_ seeds

In our study, two sets of F1 hybrids were prepared using the North Carolina mating design II [31]. One set contained 48 F1 hybrids made by 8 CMS and 6 restorer lines in Indica rice subspecies, and the other set consisted of 78 F1 hybrids made by 13 CMS and 6 restorer lines in Japonica rice subspecies. These parental lines are widely utilized for the commercial production in three-line hybrid rice in China. Dry seeds of the 33 parents were sown in the first week of May 2014 on the seedling nursery in paddy field at the Jiangpu Experimental station (32°07′N, 118°64′E), Nanjing Agricultural University, Nanjing, China. Thirty days after sowing, the seedlings were transplanted by hand into the paddy field with each plot containing 5 rows with 8 plants per row at a space of 20 cm × 20 cm. At heading stage, the spikelets of CMS plants were cut one third using scissors by hand before floret flowering, pollinated with restorer pollen and covered with Kraft paper bag (Fig. 6a). Twenty five days after pollination, the F1 seeds were harvested (Fig. 6b), dried by air, threshed by hand and stored in room temperature. The F1 hybrids’ seeds and their parents (restorer lines, and maintainer lines instead of CMS lines) were nursed in the first week of May 2015. Thirty days later, the seedlings were transplanted into the paddy field with one seedling per hill using a randomized complete block design and three replications. Each plot contained 5 rows with 8 plants per row at a space of 20 cm × 20 cm,. Field management practices were implemented according to the local standards. After the lowest grain on main stem panicle in all plants within a plot became yellow, all the panicles of randomly-selected 6 plants within the plot were harvested by hand, put them into a Nylon mesh bag, dried them under the sun, and threshed by hand. The dried grains were stored three months at room temperature for stabling the physicochemical traits of rice qualities.

### Traits measurements

Brown rice rate is calculated using the following formula:

$$ \mathrm{Brown}\ \mathrm{rice}\ \mathrm{rate}\ \left(\%\right)=\frac{\ \mathrm{Brown}\ \mathrm{rice}\ \mathrm{weight}}{\ \mathrm{Unhulled}\ \mathrm{rice}\ \mathrm{weight}} \times 100 $$

**Fig. 7:**
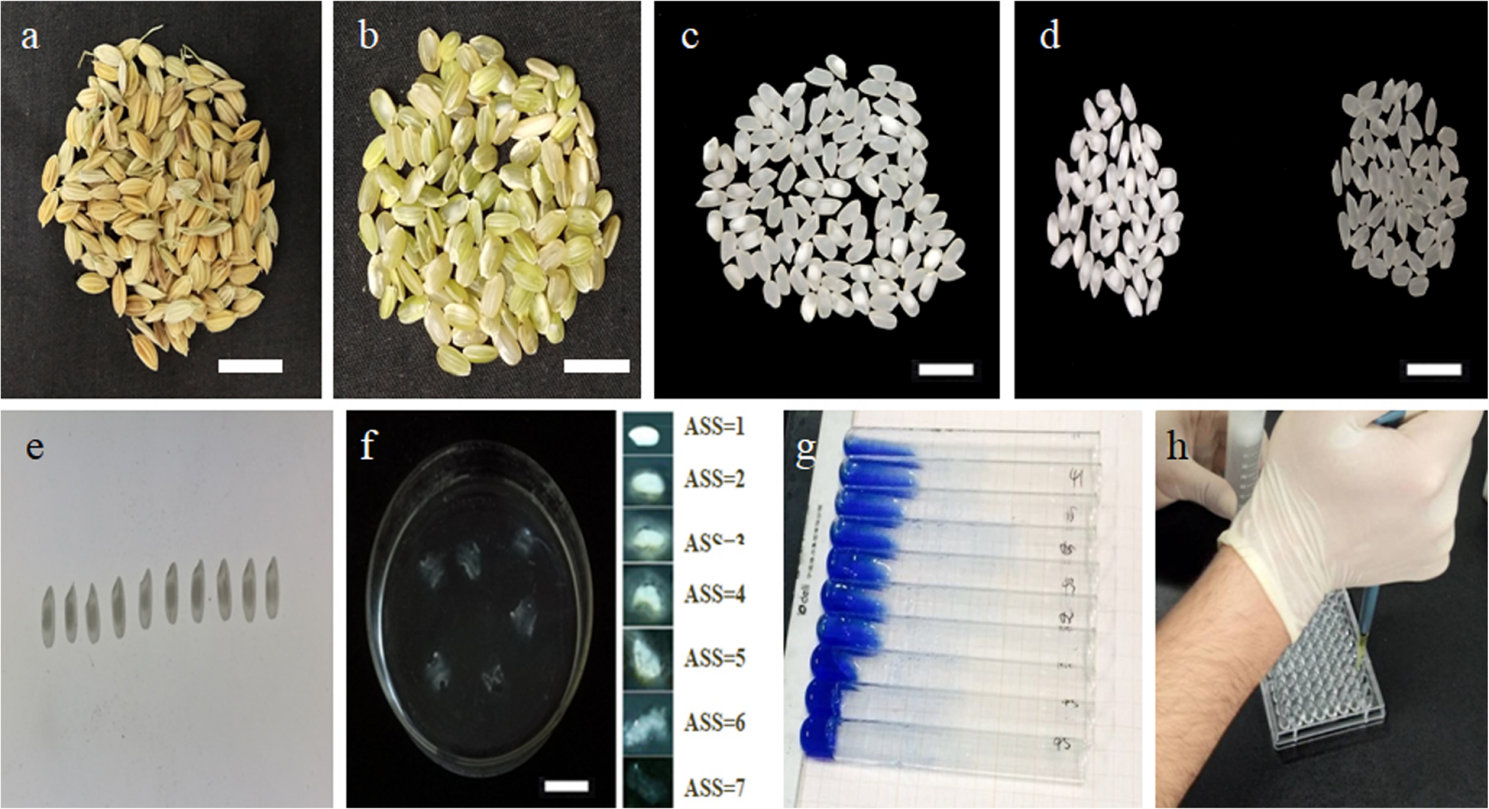
Partial photos of the experiment operation process of quality traits measurements. **a**. Unhulled rice grains. **b**. Brown rice grains. **c**. Head rice grains. **d**. Chalky grains. **e**. Chalky area size. **f**. Alkali spread score. **g**. Gel consistency. **h**. Amylose content

Where, brown rice or husked rice was obtained by removing the husk of raw rice (Fig. 7a, b) by a brown rice machine (JE0826, Zhejiang Taizhou grain Instrument Factory).

Milled rice rate is calculated by using the formula:

$$ \mathrm{Milled}\ \mathrm{rice}\ \mathrm{rate}\ \left(\%\right)=\frac{\ \mathrm{Milled}\ \mathrm{rice}\ \mathrm{weight}}{\mathrm{Unhulled}\ \mathrm{rice}\ \mathrm{weight}}\times 100 $$

Where, milled rice was obtained by removing all or part of the bran and embryo from the husked rice grains using the Laboratory Huller (JMNJG3, Zhejiang Taizhou grain Instrument Factory).

Head rice rate is calculated by using the formula:

$$ \mathrm{Head}\ \mathrm{rice}\ \mathrm{rate}\ \left(\%\right)=\frac{\mathrm{Intact}\ \mathrm{grains}\ \mathrm{weight}}{\mathrm{Unhulled}\ \mathrm{rice}\ \mathrm{weight}}\times 100 $$

Head rice indicates to milled rice grains whose length is greater than or equal to three quarters of the average length of the intact grain (Fig. 7c).

### Percentage of chalky grains, chalky area size and chalkiness degree

Grain chalkiness was assessed by a visual rating of the chalky percentage of the grain (Fig. 7d,e) based totally on the standard Evaluation System (SES). Selection, segregation and weighing the chalky grains was performed. The test method of PCG, CAS and CD were in accordance to the method of Han et al. [10] and Hong [12]. The following formula was used to calculate the percentage of chalky grains:

$$ \mathrm{Percentageof}\kern0.5em \mathrm{Chalkygrains}=\frac{\mathrm{Chalkygrainsnumber}}{\mathrm{Headrice}\begin{array}{c}\mathrm{number}\\ {}\kern0ex \mathrm{tested}\end{array}}\times 100 $$

### Gelatinization temperature

The GT was indirectly estimated as the alkali spreading score (ASS) according to Little et al. [17] with slightly modified. Briefly, 6 grains of intact milled white rice of each accession were put in a petri dish of 60 mm in diameter and add 10 ml of 1.7% KOH water solution. The samples were separated from each other by using the forceps and incubated at 30 ± 0.5 °C for 23 h to allow spreading of the grains. The spreading score of the grains was recorded through visual assessment according to the description of Jennings et al. [14]. ASS values were recorded as grade 1 to grade 7, according to the appearance of the endosperm and the degree of dispersion. Endosperm that was unaffected were recorded 1, and that were disappeared completely (Fig. 7f) were recorded 7, ASS is inversely proportional to GT. There are three classes of gelatinization temperature: the ASS from 1 to 3 grades is high GT (> 75 °C), from 4 to 5 grades is intermediate GT (70–74 °C), and from 6 to 7 grades is low GT (< 70 °C).

### Gel consistency

The GC was determined following the method of Cagampang et al. [4]. Briefly, 100 mg of rice flour about 12% of the moisture content was weighed in a test tube, to which 0.2 ml of 95% ethanol containing thymol blue was added and gently shaken to forestall cluttering of the powder during gelatinization. Two milliliters of 0.2 mol/L KOH was added and thoroughly shaken. The tubes were covered with the glass marbles and boiled in a water bath to reflux for 8 min. After cooling down at room temperature for 5~10 min, the tubes were placed on ice for 20 min, and then placed horizontally on the surface of the table for 1 h. The length of the gel (mm), that is, the distance from the bottom of the tube to the front of the gel migration, is a measurement of GC (Fig. 7g). The longer the gel is, the softer the GC is.

### Amylose content

The AC of head rice grains was measured by using the automatic microplate spectrophotometer followed the method described by Zhu et al. [41]. Briefly, each 50 mg of test sample flour was taken in a test tube with scale, added 0.5 ml of 95% ethanol and 1.5 ml of 3 mol/L NaOH, respectively. The tube was placed overnight (12-16 h) in 30 °C thermostat after fully skimmed then shaken lightly with distilled water to 40 ml. 10 μl mixture was added on the ELISA plate with 96 holes (Fig. 7h), remaining reaction solution consisted of 2 μl of 1 mol/L acetic acid and 3 μl of 2% I_2_-KI, finally adding distilled water 185 μl, so that each hole volume to 200 μl. The remaining step is setting the wavelength of 620 nm, the microplate spectrophotometer automatically recorded the optical density (OD) value, which was showed in the computer monitor. The content of amylose was calculated by a standard curve.

### Calculation of effect values of GCA

According to the statistical model [32], the obtained phenotypic data of the hybrids have been subjected to analysis of variance (ANOVA) by using excel software (2007). The following equations were used to analyze the effect values of GCA in 9 quality-related traits for the 33 parents [40].

$$ {v}_{ik}=\mu +{g}_i+{\varepsilon}_{ik} $$

where, *μ* =mean,

*g*_*i*_= general combining ability effect of *i*_*th*_ genotype,

*ε*_*ik*_ = error term associated with *i*_*th*_ genotype and *k*_*th*_ replication.

Significance of genotype differences was tested using the method of least significant difference (LSD) at α = 0.01 probability level.

### Construction of SNPLDBs

The SNP data of this study were obtained from Zaid et al. [39], and are available at NCBI under accession number SRR7250921. The missing genotypes in the sequence data were precisely predict through the fastPHASE software [35]. Then, The linkage disequilibrium (LD) blocks were defined by Haploview software [2]. The confidence intervals block partitioning approach was used with default settings, except that 200 kb and 0.01 were the maximum distance and minimum major allele frequency (MAF), respectively [7]. To construct SNPLDB, the SNPs inside the block have been grouped into a single marker with haplotype as its alleles. Each constructed SNPDB was tested in association analysis after treating it as a marker. The markers of constructed SNPLDB were characterized in detail by using Power Marker software (v3.25) through calculating their MAF, genetic diversity and polymorphism information content (PIC) [20].

### Association analysis

In order to identify significantly associated SNPLDBs with the effect values of GCA for the parents of hybrid rice, the single-factor analysis of variance (ANOVA) was used to test the association between the effect values of GCA for 9 quality-related traits and the constructed SNPLBDs [27]. The linear model is

$$ {y}_{ij}=\mu +{a}_i+{\varepsilon}_{ij} $$

Where y_*ij*_ is the i_*th*_ allele j_*th*_ observation at the SNPLDB under testing.

μ is the mean of population,

a_*i*_ is the i_*th*_ allele effect and

ε_*ij*_ is error of random.

SAS PROC GLM (Release 9.1.3; SAS Institute, Cary, NC) was used to perform all the computation processes. The significant SNPLDBs on the chromosomal region had been selected at the least *P* value (α = 0.001 probability level) for all traits except chalky area size and amylose content which were selected at P value (α = 0.01 probability level). The coefficient of determination (R^2^) was estimated to determine the percentage of phenotypic variation explained by each associated SNPLDB marker.

## Supplementary information

**Additional file 1: Table S1.** Means of 9 quality traits in 48 Indica crosses (F2 bulks).

**Additional file 2: Table S2.** Means of 9 quality traits in 78 Japonica crosses (F2 bulks).

**Additional file 3: Table S3.** The values and scores of five levels of nine grain quality traits in Indica rice.

**Additional file 4: Table S4.** The values and scores of five levels of nine grain quality traits in Japonica rice.

## Data Availability

All data generated or analyzed during this study are included in this published article and supplementary information files. All the SNP data of this study obtained from previous studies on rice CMS in our State Key Laboratory of Crop Genetics and Germplasm Enhancement, Nanjing Agricultural University, and are available at NCBI under accession number SRR7250921.

## Abbreviations

AC: Amylose content
BRR: Brown rice rate
CAS: Chalky area size
CD: Chalkiness degree
CMS: Cytoplasmic male sterile
GC: Gel consistency
GCA: General combining ability
GT: Gelatinization temperature
HRR: Head rice rate
MRR: Milled rice rate
PCG: Percentage of chalky grains
SNP: Single nucleotide polymorphism
SSR: Simple sequence repeats
